# Transcriptomic Data Analysis Reveals a Down-Expression of *Galectin-8* in Schizophrenia Hippocampus

**DOI:** 10.3390/brainsci11080973

**Published:** 2021-07-23

**Authors:** Maria Cristina Petralia, Rosella Ciurleo, Alessia Bramanti, Placido Bramanti, Andrea Saraceno, Katia Mangano, Maria Catena Quattropani, Ferdinando Nicoletti, Paolo Fagone

**Affiliations:** 1 Department of Clinical and Experimental Medicine, University of Messina, 98122 Messina, Italy; m.cristinapetralia@gmail.com (M.C.P.); maria.quattropani@unime.it (M.C.Q.); 2 IRCCS Centro Neurolesi “Bonino-Pulejo”, 98124 Messina, Italy; rossella.ciurleo@irccsme.it (R.C.); placido.bramanti@irccsme.it (P.B.); 3 Department of Medicine, University of Salerno, 84084 Salerno, Italy; abramanti@unisa.it; 4 Department of Biomedical and Biotechnological Sciences, University of Catania, 95123 Catania, Italy; andreasara96@gmail.com (A.S.); kmangano@unict.it (K.M.); ferdinic@unict.it (F.N.)

**Keywords:** schizophrenia, hippocampus, galectins, *LGALS8*

## Abstract

Schizophrenia (SCZ) is a severe psychiatric disorder with several clinical manifestations that include cognitive dysfunction, decline in motivation, and psychosis. Current standards of care treatment with antipsychotic agents are often ineffective in controlling the disease, as only one-third of SCZ patients respond to medications. The mechanisms underlying the pathogenesis of SCZ remain elusive. It is believed that inflammatory processes may play a role as contributing factors to the etiology of SCZ. Galectins are a family of β-galactoside-binding lectins that contribute to the regulation of immune and inflammatory responses, and previous reports have shown their role in the maintenance of central nervous system (CNS) homeostasis and neuroinflammation. In the current study, we evaluated the expression levels of the galectin gene family in post-mortem samples of the hippocampus, associative striatum, and dorsolateral prefrontal cortex from SCZ patients. We found a significant downregulation of *LGALS8* (*Galectin-8*) in the hippocampus of SCZ patients as compared to otherwise healthy donors. Interestingly, the reduction of *LGALS8* was disease-specific, as no modulation was observed in the hippocampus from bipolar nor major depressive disorder (MDD) patients. Prediction analysis identified TBL1XR1, BRF2, and TAF7 as potential transcription factors controlling *LGALS8* expression. In addition, MIR3681HG and MIR4296 were negatively correlated with *LGALS8* expression, suggesting a role for epigenetics in the regulation of *LGALS8* levels. On the other hand, no differences in the methylation levels of *LGALS8* were observed between SCZ and matched control hippocampus. Finally, ontology analysis of the genes negatively correlated with *LGALS8* expression identified an enrichment of the NGF-stimulated transcription pathway and of the oligodendrocyte differentiation pathway. Our study identified *LGALS8* as a disease-specific gene, characterizing SCZ patients, that may in the future be exploited as a potential therapeutic target.

## 1. Introduction

Schizophrenia (SCZ) is a severe psychiatric disorder characterized by a diverse set of clinical manifestations that include cognitive dysfunction, disorganized thinking, decline in motivation, and psychosis [1]. The global prevalence of SCZ is ~1%, and the incidence is approximately 1.5/10,000 people [1]. Usually, the onset of SCZ is observed earlier in men, developing SCZ at age 18–25, while the onset in women is between 25 and 35 years [2]. To date, the etiology of SCZ is still elusive, and it is believed that both genetic and environmental factors may play a role, by inducing alterations in the dopaminergic, glutamatergic, serotonergic, and gamma-aminobutyric acid neurotransmitter systems [3]. Polymorphisms in several genes have been identified as risk factors [4,5]. The Genome-Wide Association Studies (GWAS) catalog currently reports 3831 genomic associations for 105 SCZ-related traits (https://www.ebi.ac.uk/gwas/; accessed on 25 May 2021). Cortical and subcortical alterations have been described in SCZ patients, which include morphological, biochemical, and physiological aberrations [3].

A growing body of data suggests that immunologic factors exert a role in the etiology and progression of SCZ (reviewed in [6]). Significantly, increased serum levels of interleukin (IL)-6 and of the chemokine CCL-11, as well as increased levels of IL-1 receptor antagonist and serum IL-2 receptors play a role (reviewed in [6]). In vitro production of INF-γ and IL-4 by SCZ peripheral blood mononuclear cells (PBMCs) was found to be significantly different from controls. In first-episode psychosis patients but not in unaffected siblings, significantly increased pro- and anti-inflammatory cytokines, i.e., IL-1β, IL-6, TNF-α, IL-10, and TGF-β, were observed, as compared to controls [7]. Additionally, siblings presented decreased IL-1β, when compared with patients and controls [7]. Along the same lines, leukocyte m-RNA levels of IL-1α, IL-6, and TNF-α, but not IL-1β and IL-8, were significantly increased in first-episode psychosis patients, in comparison to healthy controls, matched for age, gender, ethnicity, and body mass index [8]. Moreover, antipsychotic-naïve SCZ patients were found to have significantly higher plasma levels of IL-6 and lower levels of IL-17 and IFN-γ as compared with healthy controls [9]. Another study also showed that IL-6, IL-8, IL-10, and TNF-α positively correlated with negative psychotic symptoms, and that augmented serum levels of IL-8 are associated with poor responses to antipsychotic treatment [10].

Moreover, a significantly higher percentage of SCZ patients, as compared to controls, tested positive for anti-cardiolipin auto-antibodies (reviewed in [6]). Interestingly, positive symptoms of SCZ were associated with high levels of anti-Nerve Growth Factor (NGF) auto-antibodies, while negative symptoms were associated with higher LE activity (reviewed in [6]). Other auto-antibodies, such anti-platelet, anti-muscarinic acetylcholine-receptor (mAChR), and anti-α-7 subunit of the acetylcholine nicotinic receptor (α7AChNR), were more frequently observed in SCZ patients than controls. Finally, a significant genome association was found between SCZ and the MHC locus on chromosome 6, as well as the IL-2 single nucleotide polymorphism (SNP) of the TT genotype and the IL-4 SNP of the CC genotype (reviewed in [6]).

Galectins are a family of β-galactoside-binding lectins that take part in several biological processes, such cell adhesion and motility, autophagy, proliferation, angiogenesis, cell signaling, immune responses, and inflammation [11]. Galectins are small soluble proteins that contain either one or two carbohydrate recognition domains (CRDs). Galectins are present in both the cytosol and nucleus, as well as in the extracellular compartment, as they are secreted by a non-classical secretion process, which bypasses the Golgi complex [11]. Increasing data suggest that galectins, such as Galectin-1 and Galectin-3, are involved in the regulation of CNS homeostasis and neuroinflammation by regulating microglial activation, dampening neurodegeneration, and favoring neuroprotection [12]. Galectin-1, Galectin-3, Galectin-4, Galectin-8, and Galectin-9 are highly expressed in the brain [13]; however, to date, most studies on CNS function and protection have been dedicated to Galectin-1 and Galectin-3 [14].

In the present study, we investigated the expression levels of the galectin gene family members in post-mortem samples of different brain regions of SCZ patients, as compared to matched control and bipolar and major depressive disorder (MDD) patients as well. We found a significant downregulation of *LGALS8* (*Galectin-8*) in the hippocampus of SCZ patients, in comparison to otherwise healthy donors. Interestingly, the down-expression of *LGALS8* was disease-specific, as no modulation was observed in the hippocampus from the bipolar nor MDD hippocampus. Moreover, ontology analysis of the genes negatively correlated with *LGALS8* expression identified an enrichment of the NGF-stimulated transcription pathway and of the oligodendrocyte differentiation pathway.

## 2. Materials and Methods

### 2.1. Dataset Selection and Analysis

The NCBI Gene Expression Omnibus (GEO) database (http://www.ncbi.nlm.nih.gov/geo/, accessed on 2 March 2021) was used to identify microarray datasets comparing the transcriptomic profiles of brain from healthy donors and SCZ patients. The GEO database was manually searched using the MeSH term (Medical Subject Headings) “schizophrenia”. The datasets were selected if they met the following inclusion criteria: (a) whole-genome transcriptomic profiling; (b) tissues collected from defined brain regions; (c) consisted of both one cohort of SCZ patients and another cohort of healthy people; and (d) species of origin “*Homo sapiens*”. Finally, the GSE53987 was selected for the analysis, as it included whole genome transcriptomic data from post-mortem samples of hippocampus, associative striatum, and dorsolateral prefrontal cortex from SCZ patients, as well as control subjects and patients suffering from bipolar disorder and major depressive disorder (MDD) [15]. The clinical data available for the patients included in the GSE53987 dataset are summarized in Table 1. Briefly, the GSE53987 dataset included 19 samples from healthy people, 15 samples from SCZ patients, 18 samples from bipolar patients, and 17 from MDD patients [15]. Data were subjected to quantile normalization, and low variance genes were excluded from the analysis. For this study, the expression levels of *LGALS1*, *LGALS2*, *LGALS3*, *LGALS3BP*, *LGALS4*, *LGALS8*, *LGALS9*, *LGALS12*, *LGALS13*, and *LGALS14* were evaluated.

### 2.2. Gene Ontology Analysis

Pathway analysis, Gene Ontology, and interactome analysis were performed using the online software Metascape [16]. The hypergeometric test and Benjamini–Hochberg *p*-value correction are used to identify significant enriched ontology terms. Metascape analysis relies on several databases, including KEGG, MSigDB, Gene Ontology, and Reactome, among the others. Enriched terms are clustered into non-redundant groups, by calculating the pairwise similarity between the enriched terms, using a Kappa-test score [16]. 

### 2.3. Gene Expression Regulation

For the identification of the transcription factors (TFs) that could regulate the expression of *LGALS8*, a list of putative TFs was first retrieved from John and Mishra (2016) [17]. The TFs mostly correlated (FDR < 0.05) to *LGALS8* expression were then clustered using the Spearman rank correlation, as similarity metrics. For the identification of miRNAs putatively regulating *LGALS8* expression, the miRWalk database was interrogated (http://mirwalk.umm.uni-heidelberg.de/, accessed on 23 July 2021). Among the predicted miRNAs, those negatively correlated to *LGALS8* expression were selected. For the analysis of the methylation pattern of *LGALS8*, the GSE89703 dataset was interrogated. The dataset included post-mortem hippocampus samples: 14 schizophrenia and 13 controls. Bisulfite converted DNA from these samples were hybridized to the Illumina Infinium 450k Human Methylation Beadchip v1.0 [18].

### 2.4. Statistical Analysis

Statistical analysis for the microarray data was performed using the LIMMA (Linear Model for MicroArray Analysis) test. An adjusted (Benjamini–Hochberg corrected) *p*-value (adj. *p*-value, FDR: False Discovery Rate) < 0.05 was considered as threshold for statistical significance. Hierarchical clustering and gene similarity matrix were calculated using the Spearman rank correlation, as similarity metrics. For the identification of the genes significantly correlated to *LGALS8*, the Pearson correlation was calculated, and the statistical significance was computed using a permutation test, with 1000 random permutations. Correlated genes were selected based on *p*-value < 0.01 and ǀrǀ > 0.7.

## 3. Results

### Expression Levels of the Galectin Gene Family Members in Different Brain Regions from Control Donors

**Figure 1:**
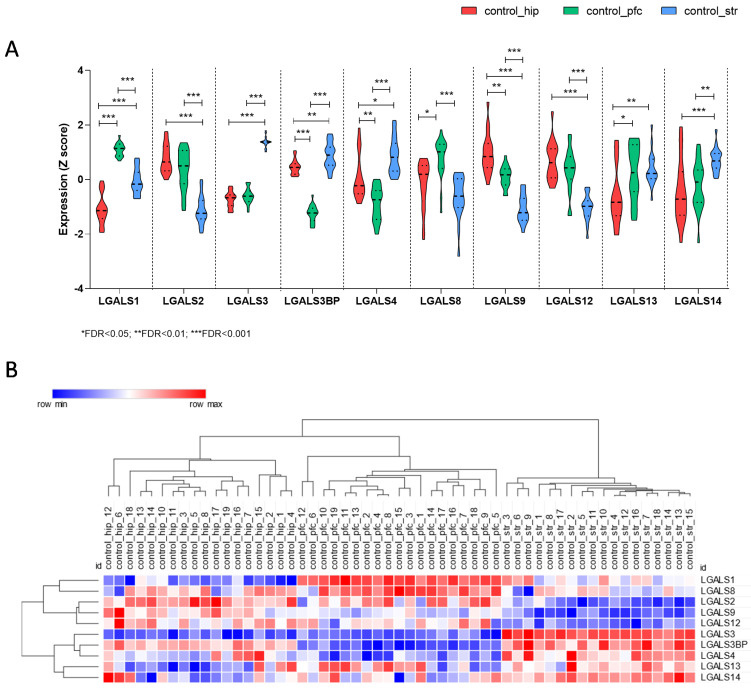
Expression levels of the galectin gene family members in the normal brain. The expression levels of the members of the galectin gene family were determined by interrogating the GSE53987 dataset. (**A**) Violin plots showing the expression levels of the galectin genes in the hippocampus, prefrontal cortex, and striatum of normal donors. (**B**) Hierarchical clustering for the expression levels of the galectin genes; the hippocampus, prefrontal cortex, and striatum of normal donors were calculated using the Spearman rank correlation as similarity metrics.

First, we wanted to determine the expression levels of the galectin gene family members in different brain areas from control donors. As shown in Figure 1, significant differences could be observed in the expression levels of the galectins in the hippocampus, associative striatum, and the prefrontal cortex (Figure 1A). In particular, the hippocampus was characterized by a down-expression of *LGALS1*, *LGALS13*, and *LGALS14*, as compared to striatum and prefrontal cortex. An over-expression of *LGALS1* and *LGALS8* was instead observed in the prefrontal cortex, as compared to the hippocampus and striatum. Significant higher levels of *LGALS3*, *LGALS3BP*, and *LGALS14*, as well as significant lower levels of *LGALS2*, *LGALS8*, *LGALS9*, and *LGALS12* characterized the associative striatum when compared to the hippocampus and the prefrontal cortex (Figure 1A). Along the same lines, hierarchical cluster analysis revealed that the hippocampus, prefrontal cortex, and striatum could be segregated by considering the expression levels of the galectin gene family members (Figure 1B).

**Figure 2:**
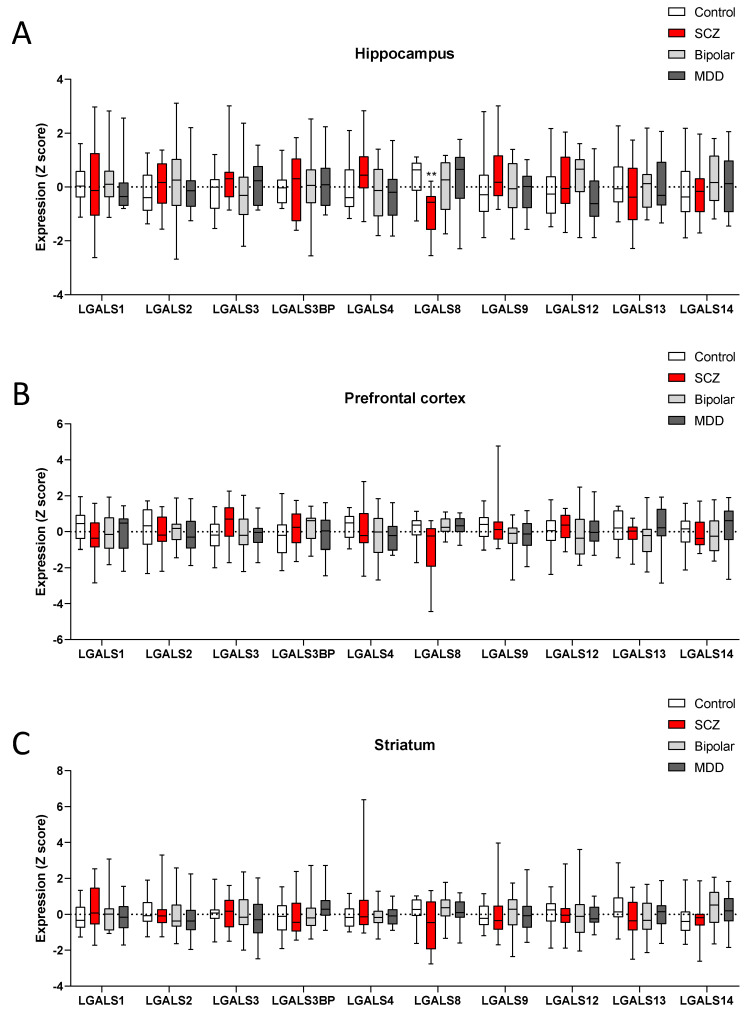
Expression levels of galectin gene family members in the SCZ brain. The expression levels of the members of the galectin gene family were determined by interrogating the GSE53987 dataset. (**A**) Expression levels of the galectin genes in post-mortem samples of the hippocampus from control donors, and SCZ, bipolar, and MDD patients. (**B**) Expression levels of the galectin genes in post-mortem samples of the prefrontal cortex from control donors, and SCZ, bipolar, and MDD patients. (**C**) Expression levels of the galectin genes in post-mortem samples of the striatum from control donors, and SCZ, bipolar, and MDD patients.

Next, we determined the relative expression levels of the different members of the galectin family in SCZ patients. As shown in Figure 2, only *LGALS8* was significantly downregulated in the hippocampus of SCZ patients, as compared to the control donors (Figure 2). Interestingly, no modulation of *LGALS8* was observed in either the bipolar or the MDD hippocampus samples (Figure 2). A trend of reduced levels of *LGALS8* was also observed in the striatum and the prefrontal cortex from SCZ patients, but statistical significance was not reached. No modulation was observed in the expression levels of all the other members of the galectin family, in either of the brain areas considered and for either the bipolar or MDD patients (Figure 2)

Since all the SCZ patients were under antipsychotic treatment, we aimed to determine whether the modulation in *LGALS8* expression was related to drug exposure rather than to the disease. To this aim, we interrogated the GSE66277, which included whole-genome expression data from male Sprague–Dawley rats chronically treated with either haloperidol, risperidone, or vehicle. No modulation of *LGALS8* was observed in the hippocampus of the treated rats (Supplementary Materials Figure S1).

**Figure 3:**
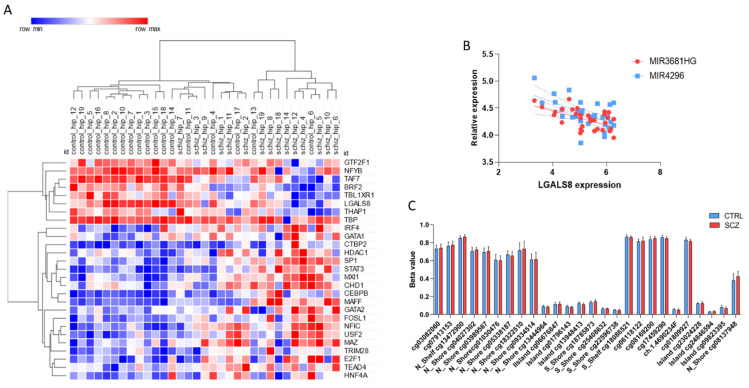
Regulation of *LGALS8* expression in SCZ brain. (**A**) Hierarchical clustering of the transcription factors predicted for *LGALS8* in the SCZ hippocampus, as determined in the GSE53987 dataset. (**B**) Correlation between *LGALS8* expression and *MIR3681HG* and *MIR4296*, as determined in the GSE53987 dataset. (**C**) Methylation levels of the LGALS8 gene in the hippocampus from SCZ patients and control donors, as determined in the GSE89703 dataset.

Next, we wanted to determine the mechanisms of regulation of *LGALS8* expression in the SCZ hippocampus. As shown in Figure 3A, among the putative TFs regulating *LGALS8* expression, TBL1XR1, BRF2, and TAF7 were the most positively correlated TFs (FDR < 0.05) (Figure 3A). Moreover, among the miRNAs predicted for *LGALS8*, two of them, i.e., MIR3681HG and MIR4296, were negatively correlated with *LGALS8* expression, suggesting a role for epigenetics in the regulation of *LGALS8* levels (Figure 3B). On the other hand, no significant differences were observed in the methylation levels of *LGALS8* between SCZ and matched control donor hippocampus (Figure 3C).

**Figure 4:**
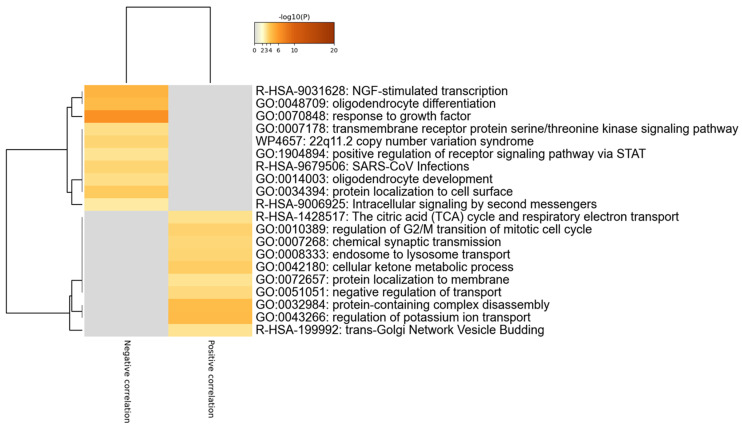
Gene Ontology and pathway analysis for the genes most correlating with *LSGALS8* in SCZ hippocampus. Genes significantly correlated to *LSGALS8* in SCZ hippocampus were identified by calculating the Pearson correlation, and the statistical significance was computed using a permutation test, with 1000 random permutations. The graph represents the hierarchical clustering of the most enriched biological processes and gene ontologies among the genes significantly correlated to *LGALS8*, as determined using the web-based utility, Metascape.

**Figure 5:**
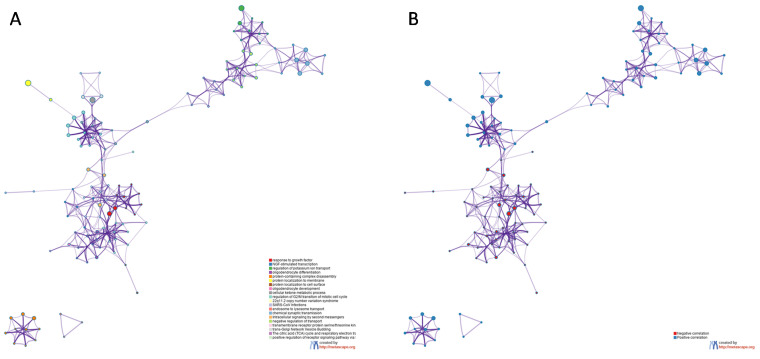
Enrichment analysis for the genes most correlating with LSGALS8 in SCZ hippocampus. Genes significantly correlated to *LSGALS8* in SCZ hippocampus were identified by calculating the Pearson correlation, and the statistical significance was computed using a permutation test, with 1000 random permutations. (**A**) Network showing the interconnection among the most enriched terms. Each term is represented by a circle node, with a size proportional to the number of genes that fall into each term, and the color representing the cluster identity; (**B**) the same network with the nodes colored based on whether the specific enriched term is enriched by either the genes positively (blue) or negatively (red) correlated to *LGALS8*.

Finally, to gain insights into the biological meaning of *LGALS8* modulation in the SCZ hippocampus, we first identified the genes significantly correlated, both positively and negatively, to *LGALS8* expression, and Gene Ontology and pathway analysis were performed. In total, 115 genes were negatively correlated, while 177 genes were positively correlated to *LGALS8* (*p*-value < 0.01 and ǀrǀ > 0.7). Several enriched terms were found for both the positively and negatively correlated genes (Figure 4 and Figure 5). In particular, R-HSA-9031628 (NGF-stimulated transcription), GO:0048709 (oligodendrocyte differentiation), and GO:0070848 (response to growth factor) were found to be enriched among the genes negatively correlated to *LGALS8*, while GO:0043266 (regulation of potassium ion transport), GO:0032984 (protein-containing complex disassembly), and GO:0042180 (cellular ketone metabolic process) were found to be enriched among the genes positively correlated to *LGALS8* (Figure 4 and Figure 5).

## 4. Discussion

Dysfunction in the prefrontal cortex, hippocampus, and associative striatum has been described for several psychiatric diseases, including SCZ [15,19,20,21,22]. Previous reports have shown differential expression of multiple genes across brain regions in SCZ patients, as compared with control donors [15,19]. In these studies, the hippocampus has shown the highest number of transcriptional changes in SCZ [15,19]. The most striking result of these studies is the consistent upregulation of inflammatory pathways in the brain of SCZ subjects [23]. Interestingly, on the other hand, bipolar and MDD samples showed very little enrichment of the same inflammatory pathways [15,19].

The finding of the inflammatory dysregulation underlying SCZ has prompted significant interest in testing the immunomodulatory potential of psychotropic medications. Unfortunately, discordant results have been obtained among studies, which dampens the possibility of reaching conclusive results [24]. Additionally, clinical trials on the effects of anti-inflammatory drugs have shown mixed benefit in patients with SCZ [25]. This is likely due to the low passage of most current anti-inflammatory drugs across the blood–brain barrier (BBB). Hence, strategies to enhance the BBB permeation of drugs aimed at reducing neuroinflammation may represent in the future a viable therapeutic strategy for treating SCZ. It is also likely that earlier intervention may be more effective, as the persistence of inflammation in the adult SCZ brain could cut down the possibility of producing measurable improvements, when prolonged inflammatory processes cause irreversible structural alterations in the brain circuitry.

The role of inflammation in the pathogenesis of SC is supported by animal models showing that prenatal immune activation determines a series of brain alterations, such as reduction in parvalbumin interneurons, disrupted working memory, and alteration in both NMDA and dopamine signaling, which are relevant to SCZ [26,27]. Moreover, animal exposure to an acute prenatal immune activation is associated with persistent increases in cytokine production in the frontal cortex and hippocampus of young adult animals [28,29].

In accordance with the role of inflammation in SCZ (reviewed in [6]), it was shown that following treatment of the acute illness, IL-6 levels significantly decreased in SCZ patients, along with an increase in sIL-2R [30]. In another meta-analysis, brain-derived neurotrophic factor (BDNF) increased upon treatment in SCZ along with a decrease in serum IL6 and TNF-α concentrations [31]. Additionally, after risperidone treatment, IL-6, IL-10, TNF-α, and IL-4 decreased significantly [32]. No significant differences were found between the post-treatment cytokine levels in first episode psychosis patients and in healthy controls, suggesting that these patterns of altered cytokine levels may represent a marker of first episode psychosis [32]. In addition, there was a significant negative association between the risperidone-induced changes in IL-10 and the negative symptoms [32].

In the present study, by making use of a publicly available transcriptomic dataset, we observed a significant downregulation of *LGALS8* in the hippocampus from SCZ patients, which negatively correlated to genes involved in the regulation of oligodendrocyte development and response to NGF. The use of whole-genome expression databases has been largely exploited by our group and others [33,34,35,36,37] for the characterization of pathogenic pathways and to identify therapeutic targets for a variety of disorders, such as autoimmune diseases [38,39,40,41,42,43,44,45,46] and cancer [40,47,48] and has allowed pathogenic pathways [49,50,51,52] and potential therapeutic targets [53,54,55,56,57] to be characterized.

Galectin-8 belongs to the tandem-repeat class of galectins characterized by two CRDs, which are linked by peptides of varying length [58,59]. Unlike the other galectins, Galectin-8 specifically binds the α2,3-sialylated glycans via its N-terminal CRD [58,59]. Intracellularly, Galectin-8, by binding the cytosolic exposed luminal glycans of damaged endosomes and lysosomes to the NDP52 autophagy adaptor, promotes their autophagic removal [60]. This function represents a defense mechanism against infections and seems to protect against the aggregation of the tau protein, involved in the pathogenesis of Alzheimer’s disease [60,61,62,63]. In the extracellular compartment, Gal-8 binds β1-integrins and activates the downstream ERK1/2 and PI3K/AKT signaling pathways [64,65,66]. Pardo and colleagues have shown in vitro that Galectin-8 protects hippocampal neurons against stress, including nutrient deprivation, oxidative stress, β-amyloid oligomers exposure, and glutamate-induced excitotoxicity [67]. In culture, primary hippocampal neurons secrete Galectin-8, and, in vitro, anti-Galectin-8 auto-antibodies from Systemic Lupus Erythematosus patients affect neuron survival [67]. Finally, higher levels of apoptosis are observed in the hippocampus from *Galectin-8* knockout mice, upon local injection of hydrogen peroxide, suggesting the neuroprotective effect of Galectin-8 in the hippocampus [67]. Hence, it is possible that the down-expression of *LGALS8* observed in the postmortem SCZ hippocampus is not a downstream result of pathological processes but could be itself a mediator of the disease.

It should be noted that the reduction of *LGALS8* was disease- and region-specific, as no modulation was observed in the hippocampus from bipolar nor MDD patients, nor in the associative striatum and prefrontal cortex. This is in line with the observation that the greatest regional burden of transcriptional changes was different among SCZ, bipolar, and MDD subjects [15]. In particular, it was observed that MDD samples showed more differentially expressed genes in the striatum and hippocampus than in the prefrontal cortex, whereas a lower number of transcripts were altered in the striatum from BD patients [15]. Additionally, the most dramatic alterations were found for SCZ subjects in all of the brain regions studied, when compared to BD and MDD samples. Indeed, the hippocampus showed the highest number of transcriptomic changes in SCZ, while the lower number of differentially expressed genes was detected in the prefrontal cortex [15,68,69]. It is also interesting to note that among the altered biological pathways underlying SCZ [70], several of them were enriched by the DEGs significantly correlated to *LGALS8* expression, including cell cycle proliferation, survival, and ion channel homeostasis.

The analysis of the hippocampus from rats chronically administered with antipsychotic drugs showed no modulation in *LGALS8* expression. These data suggest that the modulation of *LGALS8* in SCZ may not be dependent on medication and could instead independently underlie SCZ pathology. However, there are some points that need to be addressed: the analysis of the rat hippocampus was performed on animals treated with either risperidone or haloperidol. Hence, we may have missed the effect of other antipsychotic medications and also the effect of their combined administration, which is common for psychiatric patients. Moreover, the medications were dosed to healthy rats, without neurological conditions resembling SCZ pathology. Finally, differences in the metabolism between rats and humans could have biased our observation. One final limitation of this study is that bulk transcriptomic data were used for this study. Hence, future studies will need to focus on cell-specific changes, which may help to clarify the cell populations within the hippocampus undergoing the modulation of *LGALS8*, and the consequences to the other cellular populations. Additionally, the enrollment of larger cohorts of patients will help to better correlate the observed transcriptional change to the symptoms of SCZ and its progression.

## 5. Conclusions

Overall, with the present study, we are providing a comprehensive view of the expression levels of the different members of the galectin gene family in different brain areas of patients with SCZ. The significant reduced levels of *LGALS8* observed in the hippocampus from SCZ patients appears to be a disease-specific hallmark of SCZ. However, whether this modulation of *LGALS8* is a driver of the disease or a consequence of genetic/epigenetic factors remains to be determined. Upon the precise identification of the *LGALS8*-related pathways underlying SCZ pathogenesis, the use of drug discovery approaches will help to design tailored pharmacological strategies that may regulate these pathways.

## Figures and Tables

**Table 1:** Clinical data of patients included in GSE53987.

	Control	Bipolar	Major Depressive Disorder	Schizophrenia
Sex (M/F)	10/9	10/9	10/9	10/9
Race (Caucasian/Afro-American)	18/1	19/0	18/1	13/6
Age (years)	48.1 ± 10.6	46.3 ± 9.5	45.2 ± 10.1	45.1 ± 8.5

## Data Availability

All data are available from the GSE53987 dataset, freely available from the Gene Expression Omnibus (GEO) database.

## Supplementary Materials

The following are available online at https://www.mdpi.com/article/10.3390/brainsci11080973/s1, Figure S1: Effect of chronic anti-psychotic treatment on *Galectin-8* expression in rat hippocampus.
