# Supplementary material for: Transcriptomic Data Analysis Reveals a Down-Expression of Galectin-8 in Schizophrenia Hippocampus
Source: Brain Sci. 2021 Jul 23;11(8):973. doi: 10.3390/brainsci11080973 (PMC8392448; doi:10.3390/brainsci11080973)
Supplement: Supplementary file 1 [file brainsci-11-00973-s001.zip › brainsci-1282543-supplementary.pdf]

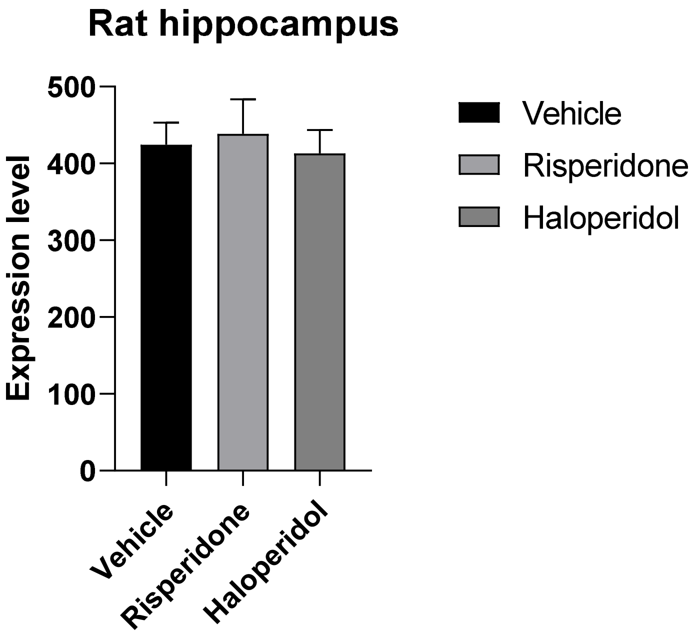

Figure S1. Effect of chronic anti-psychotic treatment on Galectin-8 expression in rat hippocampus. The GSE66277 included whole-genome expression data from male Sprague-Dawley rats treated with either 0.25 mg/kg/d haloperidol, 5 mg/kg/d risperidone or vehicle (1% acetic acid in water) for 21 days (n = 5 rats per group) (DOI: 10.1038/s41398-019-0492-8). For the datasets, the Affymetrix Rat Genome 230 2.0 Array was used. The LIMMA (Linear Model for Microarray Analysis) algorithm was used to assess differences among the groups. An adjusted (Benjamini-Hochberg corrected) p-value < 0.05 was considered to be statistically significant.
